# Detection of ABO Blood Groups From Dentin and Pulp by Using the Absorption-Elution Technique: A Forensic Cross-Sectional Study Among the Population of the Al Jouf Province, Saudi Arabia

**DOI:** 10.7759/cureus.54340

**Published:** 2024-02-16

**Authors:** Krishna A Rao, Raqiyah H Alrayes, Wafa Faisal Alshammari, Hanin Madallah AL-anazi, Elham Ali Omar Kamal, Santosh Patil

**Affiliations:** 1 Department of Preventive Dentistry, College of Dentistry, Jouf University, Sakaka, SAU; 2 Department of Oral Medicine and Radiology, Chhattisgarh Dental College & Research Institute, Rajnandgaon, IND

**Keywords:** pulp, dentin, absorption-elution technique, abo grouping, forensic odontology

## Abstract

Background and objective

Human teeth have a significant forensic importance. As they are the hardest of all human tissues, they are not just chemically stable but also their characteristics are maintained for a long time after death even in the most harsh environmental conditions. Despite the advances made in DNA analysis, fingerprinting, etc., ABO blood grouping still plays a significant role in the forensic practice in the field of personal identification, paternity disputes, and several other scenarios including the identification of mass disaster victims*. *The term blood groups refers to inherited antigens on the surface of red blood cells (RBCs) detected by specific antibodies. Since tooth pulp contains numerous blood vessels, blood group antigens are most certainly bound to be present in tooth pulp. Various studies have shown that blood group antigens in the pulp and dentin are preserved as long as up to two years after the demise of an individual. Absorption-elution technique has been proven to be the most sensitive, reliable, and consistent method to determine the ABO blood group from both the pulp and dentine. This study aimed to ascertain the ABO blood group from both the hard (dentin) as well as the soft tissue (pulp) of the tooth by using the absorption-elution (AE) technique and also to determine if there are any variations in identifying the blood groups from the teeth based on age and gender.

Material and methods

After obtaining due consent, we included patients of both genders aged between 16-60 years visiting the outpatient department (OPD) clinics at the College of Dentistry for periodontal or orthodontic extractions. One patient's blood type was determined by using the slide agglutination technique before any capillary blood extraction was performed; this patient served as a control. For this investigation, we used the pulp and powdered dentin samples taken from the dental extractions to test for the presence of ABO and Rhesus (Rh) factor antigens by using the AE method. The study samples were compared with the control for blood group determination. Statistical analysis was carried out using the chi-square test with Monte Carlo (MC) simulation to check for any correlation of blood grouping with age and gender.

Results

The dentin and pulp were shown to have positive blood group antigens for the ABO and Rh factors. While neither pulp nor dentin performed significantly differently in identifying the blood group antigens, pulp showed marginally higher accuracy. There was no discernible difference regarding gender or age in the dentin or pulp of any of the 45 samples studied.

Conclusions

For determining an individual's blood type and Rh factor, both the hard (dentin) and soft (pulp) tissues of a tooth are valid sources. This is particularly helpful in forensic medicine cases where teeth are the only remains that can be viably used to find out a person's identity.

## Introduction

In the legal domain, the role of forensic science generally involves the expert evidence given by a specialist in the field during the administration of justice in a court of law. In the field of forensic science, as per Locard’s principle, “every contact leaves its trace” [1]. Forensic identification of an individual is based on identification methodology along with presumptive or exclusion methodologies [2]. The enduring nature of human teeth renders them a widely recognized and durable biological material with significant forensic value. Since they are the most indestructible part of the human body, their properties are preserved long after death, even in the harshest of environments, and they are also chemically stable [3].

While forensic science has come a long way in terms of technological advancements, blood grouping is still very useful in many situations, such as identifying victims of mass disasters, resolving paternity claims, and analyzing DNA. Criminal suspects and those involved in paternity issues were the primary targets of ABO blood grouping investigations before DNA testing. The phrase "blood groups" is used to describe the hereditary antigens that some antibodies may detect on the surface of red blood cells (RBCs) [4]. Because they are the major, widespread, and readily detectable indicators, forensic blood group examinations have mostly focused on the ABO blood group system since its description by Karl Landsteiner around 1900 [3]. As Lattes has rightly mentioned, “the fact that belonging to a definite blood group is a fixed character of every human being and can be altered neither by lapse of time nor by recurrent disease”. Just like fingerprints, the blood group is a non-alterable primary character. One reason blood group chemicals are useful in medico-legal issues is that once an individual's blood group is determined, it stays that way forever [5].

Various bodily fluids, including saliva, amniotic fluid, perspiration, and blood, contain blood-type antigens [2]. The search for a trustworthy technique of blood typing from teeth has been ongoing among forensic specialists for decades. The tooth pulp is a sure bet to carry blood-type antigens due to the high concentration of blood vessels found in it. The dentinal tubules also contain chemicals related to blood groups, which have been extensively studied. Pulp and dentin continue to carry blood-type antigens as long as two years after a person dies, according to many studies [6]. Nevertheless, as the chances of antigen diffusion from saliva and blood diminish, the dispersion of ABO substances from the pulp cavity wall to the edges of dentin and enamel progressively decreases [6].

Forensic odontologists place a high value on blood type determination as a source of biological evidence from tooth material for the reasons mentioned above. Most forensic labs currently utilize the absorption-elution (AE) method to identify ABO blood type presence in saliva since it is the most sensitive, dependable, and consistent method. The principle of the AE test involves the absorption of blood group-specific agglutinin to the surface of a substance having blood group agglutinogens, followed by the elution of the absorbed antibody under a high temperature, and the agglutination of the blood cells against the corresponding antigens. Cold agglutination increases the agglutination intensity [7-18]. In this study, our objective was to determine the ABO blood group from both the hard and soft tissue of the tooth by using the AE technique and also to ascertain if there are any variations in the identification of the blood groups from the teeth based on age and gender.

## Materials and methods

The study was approved by the Jouf University ethical committee (approval no: 5-03-44). This experimental study included patients aged between 16 and 60 years who visited the outpatient department (OPD) clinics at the College of Dentistry for therapeutic periodontal or orthodontic extractions. Based on the feasibility assessment of the study, keeping the confidence interval (CI) at 95%, a sample size of 45 patients was calculated using analysis of variance (ANOVA): repeated measures effect size (f) = 0.25, α = 0.05 for three study groups, and three repeated measures with G’ Power software (latest version 3197; Heinrich-Heine-Universitat Dusseldorf, Dusseldorf, Germany). An extracted tooth from each patient was further sectioned into three parts, and each part was stored in different containers labeled as "under normal room temperature", "underwater", and in soil". To aid in statistical analysis, these samples were categorized as Group 1, Group 2, and Group 3, respectively.

The inclusion criteria were as follows: patients of both genders between the ages of 16 and 60 years possessing permanent teeth intended for extraction for periodontal or orthodontic purposes. the exclusion criteria were as follows: individuals below 16 or above 60 years of age; those with carious, fractured, grossly decayed, or root canal-initiated or treated teeth; and those with deciduous teeth. The research used a variety of items, including protective gear such as gloves, masks, and head coverings, as well as surgical spirit, glass slides, gauze pieces, blood lancets, and gauze pieces. Additionally, anti-coagulant solution ethylenediaminetetraacetic acid (EDTA), anti-sera A and anti-sera B, a lathe, carborundum disc, spoon excavators, straight fissure burs, a centrifuge, hot water bath, incubator, micropipette, Eppendorf tubes, containers, red blood cell suspension, normal saline, and a brightfield binocular compound microscope were employed.

The study was initiated based on the institutional ethical committee and LCBE approval. Patients were asked to freely participate in the research after they had given their informed consent in both English and Arabic. Then, after recording a short case history including pertinent medical history, a thorough clinical examination was conducted under artificial light. Teeth and gums that were deemed unsalvageable owing to periodontal disease and orthodontic treatment were chosen. Capillary blood samples procured by the finger-prick method from all 45 patients were considered controls, and the data regarding their blood group was tabulated and stored in an Excel sheet by providing a unique identification number for each patient. The samples obtained from the dentin and pulp of the extracted teeth were considered cases. Two co-investigators were allocated, one to carry out the clinical procedure and the other to carry out the laboratory procedure, and in turn, they were blinded to rule out any bias that may occur.

**Figure 1:**
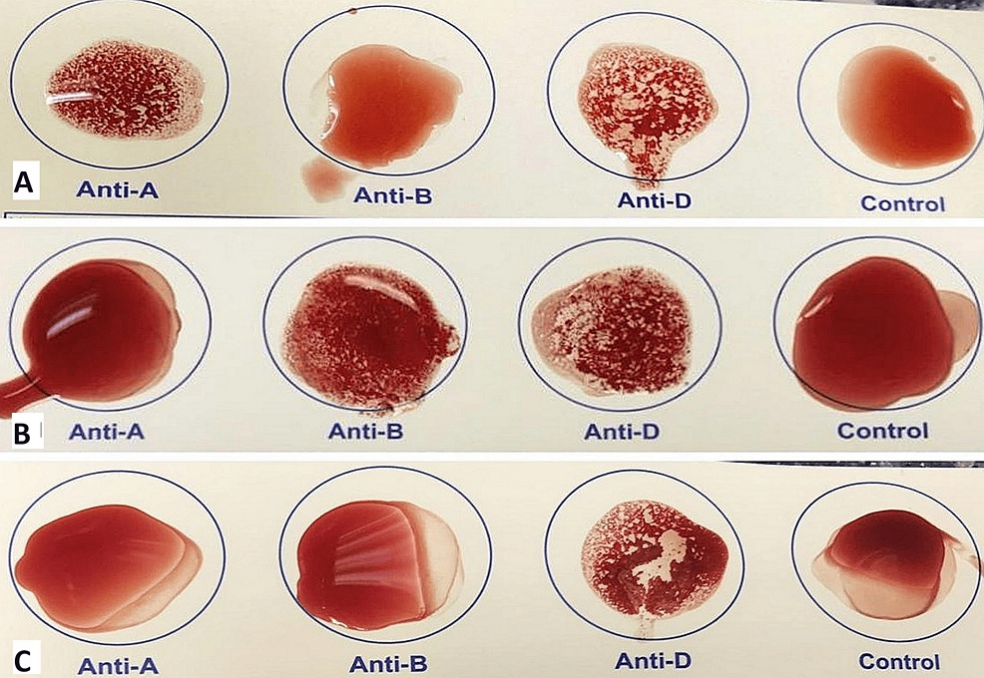
Slide agglutination method showing (A) A, (B) B, and (C) O positive blood groups respectively based on the finger-prick method

At the time of tooth extraction, blood was obtained by pricking the patient’s finger under an aseptic condition, and their blood group was determined using the slide agglutination method. Antiserum A, antiserum B, and Rhesus factor antigen were added to four drops of blood that had been deposited on the supplied placard template; the fourth drop served as a control. In cases where agglutination was seen after the addition of antiserum A, the patient's blood group was noted as A; in cases where agglutination was observed after the addition of antiserum B, the patient's blood group was noted as B. Patients were placed in the O group if no agglutination was seen; and those in whom agglutination occurred in both drops were placed in the AB group. Similarly, if agglutination was observed after adding Rhesus factor antigen, the blood group was considered positive, and if no agglutination was noticed, it was recorded as negative (Figure 1).

**Figure 2:**
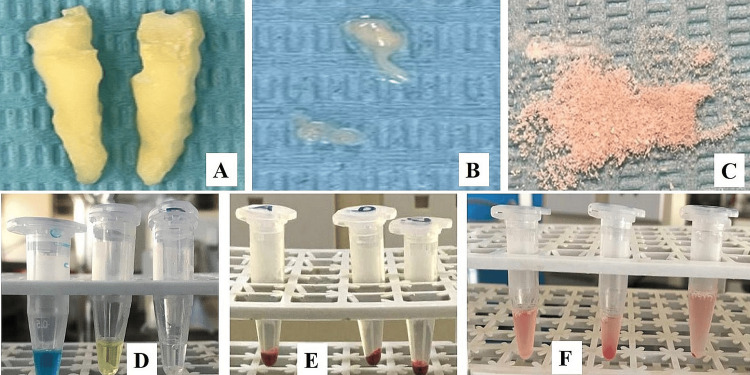
Procedure steps (A) Trimmed tooth sample. (B) Excavated pulp. (C) Pulverized dentine. (D) Pulp tissue was divided and placed in three labeled Eppendorf tubes submerged in three drops of antisera A, antisera B, and antisera D respectively. The same was done to the dentine: (E) Samples centrifuged at 1500–2000 rpm for one minute. (F) Samples after gentle shaking

Following this procedure, the extraction of the affected tooth or teeth was carried out under local anesthesia by adhering to strict aseptic protocols. After extraction, the tooth or teeth were rinsed under running water and wiped clean with sterile gauze to eliminate any remnants of saliva, blood, or other debris. As previously stated, the air-dried tooth or teeth samples were prepared by first removing the cementum and enamel using a lathe. The samples were then cut longitudinally or vertically with a carborundum disc operating at 30,000 rpm into three equal parts. Each part was then randomly assigned to one of three study groups, and each container was carefully labeled (Figure 2).

The containers with the sectioned parts of the tooth were adequately labeled with all the necessary details, including the file number, gender and age of the patient, and the date of extraction. Based on the time elapsed, as indicated by the date of extraction on the labeled bottle, each sectioned part of the tooth was then subjected to repetitive measurements at intervals of one, two, and three weeks by using the AE technique.

Absorption-elution (AE) method

**Figure 3:**
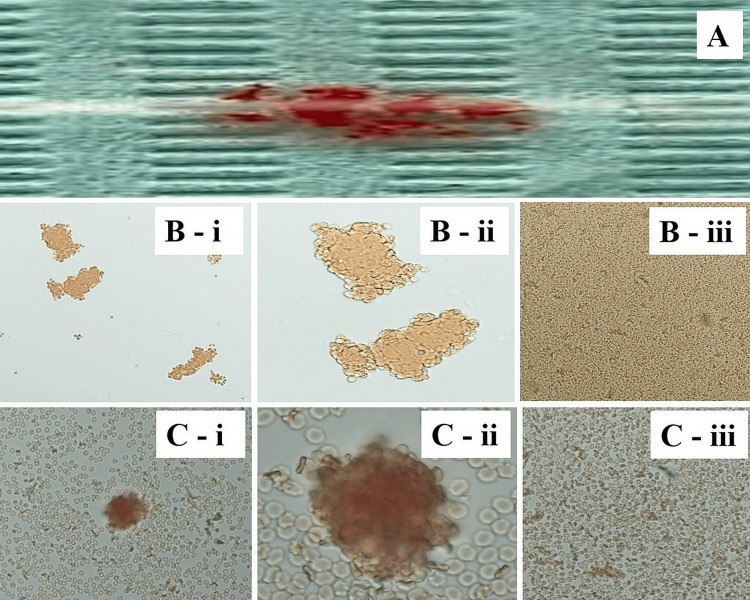
Microscopic evaluation (A) Macroscopic observation of agglutination on a glass slide in one of the samples. (B) Photomicrograph showing agglutination of red blood corpuscles (RBCs) in pulpal tissue under (i) x20,( ii) x40, and (iii) photomicrograph (x10) showing no agglutination of RBCs in pulpal tissue. (C) Photomicrograph showing agglutination of RBCs in dentin with interference from hard tissue of the tooth powder under (i) x20, (ii) x40, and (iii) photomicrograph (x10) showing no agglutination of RBCs in dentin

An access cavity was made to access the pulp tissue before grinding. To make the pulp easier to remove, a small amount of normal saline was injected into the root canal and pulp chamber of the sectioned tooth. A tooth pulp sample was extracted using a tool known as an explorer, barbed broach, or probe. A straight fissure bur was used to pulverize the remaining dentine. The pulp and the resulting dentine powder were stored in separate Eppendorf tubes. The next step was to soak the tubes for 2.5 hours at room temperature with three drops of antisera A, B, and D, respectively. Centrifuged at 3000 rpm for five minutes after three washes with cold saline solution, samples were then used for antisera removal. A pipette was used to extract the solution's supernatant. Before the Eppendorf tubes were heated in a water bath at 50-55°C for 10 minutes to elute the antibodies, two drops of saline were added. Each Eppendorf tube was promptly supplemented with a drop of newly made 0.5% red cell solution including blood types A, B, and O. To improve agglutination, the mixture was incubated at 37 °C for 30 minutes before being centrifuged at 1500-2000 rpm for one minute. Red cell agglutination was confirmed by gently shaking the Eppendorf tubes and then examining them at different magnifications by using a bright-field binocular microscope (Figure 3).

The results obtained were assessed by the primary investigator and tabulated in the Excel sheet against the control samples. A comparison between these two tabulated results was carried out by using the Chi-square test, wherein a P-value less than or equal to 0.05 indicated statistical significance. The statistical analysis was done with the help of statistical software R version 4.2.2 and Microsoft Excel (Microsoft Office Professional Plus 2016; Windows version 10).

## Results

As the same samples from the same subjects were used in all three groups, the distribution of age and gender remained the same. The age and gender distribution of the samples in all three groups is shown in Table 1.

**Table 1:** Age and gender distribution in the three groups (descriptive statistics) SD: standard deviation

Variables	Subcategory	Groups		
		Group 1 (n = 45)	Group 2 (n = 45)	Group 3 (n = 45)
Age, years	16-20	6 (13.33%)	6 (13.33%)	6 (13.33%)
	21-30	18 (40%)	18 (40%)	18 (40%)
	31-40	9 (20%)	9 (20%)	9 (20%)
	41-50	10 (22.22%)	10 (22.22%)	10 (22.22%)
	51-60	2 (4.44%)	2 (4.44%)	2 (4.44%)
	Mean ± SD, median (min, max)	31.69 ± 10.86, 28 (19, 55)	31.69 ± 10.86, 28 (19, 55)	31.69 ± 10.86, 28 (19, 55)
Gender	Male	22 (48.89%)	22 (48.89%)	22 (48.89%)
	Female	23 (51.11%)	23 (51.11%)	23 (51.11%)

The overall performance of dentin and pulp in sectioned samples in all three groups (Group 1-Group 3) as compared to the control is shown in Table 2.

**Table 2:** Overall performance of the dentin and pulp in sectioned samples as compared to control* *Based on the Chi-square test with Monte Carlo (MC) simulation. **A p-value less than or equal to 0.05 indicates statistical significance

Groups	Blood group	Total	Pulp			Dentin		
			Positive, n (%)	Negative, n (%)	P-value**	Positive, n (%)	Negative, n (%)	P-value**
Group 1 (n = 45)	Total	45	44 (97.78%)	1 (2.22%)	0.9999	37 (82.22%)	8 (17.78%)	0.7036
	A-	3	3 (100%)	0		2 (66.67%)	1 (33.33%)	
	A+	20	19 (95%)	1 (5%)		17 (85%)	3 (15%)	
	B-	2	2 (100%)	0		1 (50%)	1 (50%)	
	B+	10	10 (100%)	0		9 (90%)	1 (10%)	
	O-	1	1 (100%)	0		1 (100%)	0	
	O+	9	9 (100%)	0		7 (77.78%)	2 (22.22%)	
Group 2 (n = 45)	Total	45	42 (93.33%)	3 (6.67%)	0.7466	35 (77.78%)	10 (22.22%)	0.7011
	A-	3	3 (100%)	0		2 (66.67%)	1 (33.33%)	
	A+	20	18 (90%)	2 (10%)		16 (80%)	4 (20%)	
	B-	2	2 (100%)	0		1 (50%)	1 (50%)	
	B+	10	10 (100%)	0		9 (90%)	1 (10%)	
	O-	1	1 (100%)	0		1 (100%)	0	
	O+	9	8 (88.89%)	1 (11.11%)		6 (66.67%)	3 (33.33%)	
Group 3 (n = 45)	Total	45	42 (93.33%)	3 (6.67%)	0.6177	35 (77.78%)	10 (22.22%)	0.9735
	A-	3	3 (100%)	0		2 (66.67%)	1 (33.33%)	
	A+	20	17 (85%)	3 (15%)		16 (80%)	4 (20%)	
	B-	2	2 (100%)	0		1 (50%)	1 (50%)	
	B+	10	10 (100%)	0		8 (80%)	2 (20%)	
	O-	1	1 (100%)	0		1 (100%)	0	
	O+	9	9 (100%)	0		7 (77.78%)	2 (22.22%)	

Based on the results of the Chi-square test with Monte Carlo (MC) simulation, there was no statistically significant difference in the performance of dentin and pulp in all three groups as compared to the control, with p-values ranging from 0.99 (Group 1), 0.74 (Group 2), and 0.61 (Group 3) for pulp to 0.70 (Groups 1 and 2), and 0.97 (Group 3) for dentin (Table 2).

Tables 3 show the performance of dentin and pulp in sectioned samples as compared to the controls in terms of age.

**Table 3:** Performance of the dentin and pulp in sectioned samples as compared to control in terms of age* *Based on the Chi-square test with Monte Carlo (MC) simulation. **A p-value less than or equal to 0.05 indicates statistical significance

Groups	Age, years	Total	Pulp			Dentin		
			Positive, n (%)	Negative, n (%)	P-value**	Positive, n (%)	Negative, n (%)	P-value**
Group 1 (n = 45)	Total	45	44 (97.78%)	1 (2.22%)	0.5932	37 (82.22%)	8 (17.78%)	0.075
	16-20	6	6 (100%)	0		6 (100%)	0	
	21-30	18	18 (100%)	0		17 (94.44%)	1 (5.56%)	
	31-40	9	9 (100%)	0		6 (66.67%)	3 (33.33%)	
	41-50	10	9 (90%)	1 (10%)		6 (60%)	4 (40%)	
	51-60	2	2 (100%)	0		2 (100%)	0	
Group 2 (n = 45)	Total	45	42 (93.33%)	3 (6.67%)	0.2164	35 (77.78%)	10 (22.22%)	0.0799
	16-20	6	5 (83.33%)	1 (16.67%)		5 (83.33%)	1 (16.67%)	
	21-30	18	18 (100%)	0		17 (94.44%)	1 (5.56%)	
	31-40	9	9 (100%)	0		6 (66.67%)	3 (33.33%)	
	41-50	10	8 (80%)	2 (20%)		5 (50%)	5 (50%)	
	51-60	2	2 (100%)	0		2 (100%)	0	
Group 3 (n = 45)	Total	45	42 (93.33%)	3 (6.67%)	0.4418	35 (77.78%)	10 (22.22%)	0.1714
	16-20	6	6 (100%)	0		6 (100%)	0	
	21-30	18	17 (94.44%)	1 (5.56%)		16 (88.89%)	2 (11.11%)	
	31-40	9	9 (100%)	0		6 (66.67%)	3 (33.33%)	
	41-50	10	8 (80%)	2 (20%)		6 (60%)	4 (40%)	
	51-60	2	2 (100%)	0		1 (50%)	1 (50%)	

Again, based on the results of the Chi-square test with Monte Carlo (MC) simulation, there were no statistically significant differences in the performance of dentin and pulp in all three groups in terms of age, with p-values of 0.59 (Group 1), 0.21 (Group 2), and 0.44 (Group 3) for pulp and 0.07 (Groups 1 and 2) and 0.17 (Group 3) for dentin. However, it was observed that dentin showed lower accuracies as compared to pulp, especially in samples stored at normal room temperature and in water (Groups 1 and 2) (Table 3).

Tables 4 show the performance of dentin and pulp in sectioned samples as compared to the controls in terms of gender.

**Table 4:** Performance of the dentin and pulp in sectioned samples as compared to control in terms of gender* *Based on the Chi-square test with Monte Carlo (MC) simulation. **A p-value less than or equal to 0.05 indicates statistical significance

Groups	Gender	Total	Pulp			Dentin		
			Positive, n (%)	Negative, n (%)	P-value**	Positive, n (%)	Negative, n (%)	P-value**
Group 1 (n = 45)	Total	45	44 (97.78%)	1 (2.22%)	0.5932	37 (82.22%)	8 (17.78%)	0.075
	Female	23	23 (100%)	0		19 (82.61%)	4 (17.39%)	
	Male	22	21 (95.45%)	1 (4.55%)		18 (81.82%)	4 (18.18%)	
Group 2 (n = 45)	Total	45	42 (93.33%)	3 (6.67%)	0.2164	35 (77.78%)	10 (22.22%)	0.0799
	Female	23	21 (91.3%)	2 (8.7%)		18 (78.26%)	5 (21.74%)	
	Male	22	21 (95.45%)	1 (4.55%)		17 (77.27%)	5 (22.73%)	
Group 3 (n = 45)	Total	45	42 (93.33%)	3 (6.67%)	0.4418	35 (77.78%)	10 (22.22%)	0.1714
	Female	23	21 (91.3%)	2 (8.7%)		17 (73.91%)	6 (26.09%)	
	Male	22	21 (95.45%)	1 (4.55%)		18 (81.82%)	4 (18.18%)	

Similarly, the performance of dentin and pulp in sectioned samples in all three groups as compared to the controls concerning gender showed p-values ranging from 0.59 (Group 1), 0.21 (Group 2), and 0.44 (Group 3) for pulp to 0.07 (Groups 1 and 2) and 0.17 (Group 3) for dentin, indicating no statistically significant difference.

Nevertheless, the overall accuracy of the pulp for ABO blood group detection was 44 (97.78%) for teeth stored at normal room temperature, 42 (93.33%) for teeth stored in water, and 42 (93.33%) for teeth stored in soil. The overall accuracy of dentin for ABO blood group detection was 37 (82.22%) for teeth stored at normal room temperature, 35 (77.78%) for teeth stored in water, and 35 (77.78%) for teeth stored in soil (Table 4).

## Discussion

Forensic odontology, a subspecialty of dentistry, focuses on the use of dental evidence in criminal investigations. One possible method of identifying an individual is via blood grouping from teeth [19]. The stability of teeth has long been recognized, since they may be physically and chemically preserved for an extended duration. Identification of even severely decayed or putrefied remains is greatly assisted by the presence of blood type components in both hard and soft tissues, as well as other genetic markers like enzymes [20]. In light of this, the current study set out to examine the presence of ABO blood group and Rhesus factor antigen in the dentin and pulp of teeth that had been extracted using the AE technique. Additionally, it aimed to determine whether there is any variation in the detection of these factors based on the participant's gender and age.

Since the pulp is a soft, specialized connective tissue situated in the core of the teeth and surrounded from all sides by dental hard tissues, it is one of the most protected tissues in the human body. Studies have shown that even post-mortem changes are seen very late in the pulp [16]. Blood group antigens are always present in tooth pulp because of their extensive vascularity. Therefore, it is not too difficult to determine a person's blood type from pulp [20]. Our findings validated this, as the identification of ABO blood types and Rhesus factor antigen from pulpal tissue had an overall accuracy ranging from 42 (93.33%) to 44 (97.78%), whereas dentin samples exhibited an accuracy rate ranging from 35 (77.28%) to 37 (82.22%). Our results align with those of Ballal and David (2011), who examined 30 teeth extracted for ABO typing within six months. They discovered that blood grouping from dentin did not correlate with reference blood groups, but blood grouping from pulp had a 90% correlation. They stated that due to the significant degree of calcification, the blood group chemicals may not be readily accessible in dentin [21].

In several similar studies by Ramnarayan et al. [20], Shetty and Premalatha [16], and Smeets et al. [22] at various periods, pulp was shown to be better than dentin, and the sensitivity of the dentin and pulp decreased with the increase in periods. The most prominent current idea posits that infusion sedimentation phenomena, in conjunction with an intrinsically existent antigen, is the source of blood-type antigens in tooth-hard tissue. According to the infusion sedimentation hypothesis, saliva's water-soluble antigens will seep into the tooth's surface. Blood-type antigens are found in dentinal tubules, which is another well-known fact. According to prior research, odontoblasts are a positive source of blood group antigenicity, along with the dentinal process. Research by Imai et al. has found that blood type components are less abundant in the outer dentin than in the inner dentin, with the highest concentration found in predentin. This finding is likely related to the high organic content of these layers. The researchers found that the organic content was highest in the uncalcified predentin and the deepest dentin layer that lies next to it [23].

As per Sharma et al., the process of calcification starts in the outer dentin layer, which does not contain as much blood group material and has less organic content [24]. We found no statistically significant difference in pulp and dentin performance across the three groups when comparing them to the control samples using the Chi-square test with Monte Carlo (MC) simulation. The AE technique works by first allowing a substance with blood-group agglutinogens to absorb blood-group-specific agglutinin. Then, at a high temperature, the antibody that was absorbed is eluted, and finally, blood cells with the corresponding antigens are agglutinated. Consequently, the material's surface area becomes crucial. We found that soaking the test samples in the antisera for 2.5 hours accelerated the absorption of agglutinins. On top of that, our research was able to partially circumvent calcifications that may have prevented antigen accessibility by pulverizing hard dentin material, which expanded the surface area [25]. Per various studies by Motawei et al., [3] Aswath et al., [17] Nayyar et al., [19] Lele et al., [26] and Garg and Garg [27], when comparing the three groups to the controls in terms of age and gender, no statistically significant difference was seen between the dentin and pulp in the sectioned samples. The study by Ramnarayan et al. found that pulp and hard tissue from male teeth had a higher rate of positive results than female teeth. This was attributed to differences in the size of the teeth between the sexes and the availability of pulp for blood group identification [20].

Several factors contribute to a decline in pulp performance with age, including a smaller pool of study pulp, more pulp fibrosis with age, dehydration, pulp antigen loss, canal calcification, cell lysis, tooth contamination, procedure time, and a host of other reasons [3,20,26,28]. Conversely, our study was in agreement with Garg and Garg, who did not show any variations with increasing age [27]. If there are a few mistypes or false negatives when comparing blood groupings in our study's samples and controls, it might be due to any of the following factors: pulpal fibrosis, cell lysis, inadequate pulp volume, excessive calcification making blood group chemicals in dentin inaccessible, method failure, sample contamination, or inadequate procedure. Oral microbial flora is a host for a plethora of microorganisms, among which a sizeable population consists of gram-negative aerobes. They are prevalent not only in saliva but also on tooth surfaces [5,17]. It has been deduced from various studies that contamination of teeth by gram-negative aerobes [mainly Escherichia coli (E. coli) and Serratia marcescens (S. marcescens)] involves blood groups like agglutinogens, mimicking a B-type blood group. Studies have suggested that only one subspecies of E. coli and S. marcescens can form blood groups simulating antigens. In non-sterile containers with tooth specimens, gram‑negative bacteria grow rapidly along with yeast. This process, in turn, leads to the overgrowth of additional species. The substantial growth of such bacteria tends to obscure the pulpal blood group antigens, which can produce a negative result [6,20].

Several studies have attempted to check the feasibility of the identification of blood groups from the dentin and pulp of the teeth preserved over a longer duration, and based on their results, the blood group of an individual can be estimated from the dentin and pulp of the teeth, which are preserved for as long as 12 months to two years. However, all the studies conclude that as the time duration increased, the sensitivity and specificity of detecting the blood group antigens decreased due to the reduced antigenicity of pulp and dentin [1,3,5,20]. However, in the present study, due to time constraints, the specimens were assessed for short intervals of one to three weeks in duration. As not much time had elapsed between the study groups, no statistically significant difference in the results was obtained. Also, the study did not examine the age changes of the tooth, which is a possibility when the teeth of a small age range are compared to those of a higher age group.

## Conclusions

In this study, the presence of ABO blood group antigens and the Rh factor was examined in dentin and pulp tissues of teeth, which are stable and easy to get. Our results indicate that both dentin and pulp can be used for blood group identification, thereby narrowing down identification procedures, especially in cases where only teeth are available to confirm the identity. No significant age or gender differences were observed. Even though pulp slightly outperformed dentin at expressing ABO blood group antigens, more research is needed to validate these findings to gain deeper insights into the topic, as well as to make the method more useful for more people, since it is cheap and does not require much equipment.
